# Supplementary material for: Investigating the outcomes of virus coinfection within and across host species
Source: PLoS Pathog. 2023 May 22;19(5):e1011044. doi: 10.1371/journal.ppat.1011044 (PMC10237676; doi:10.1371/journal.ppat.1011044)
Supplement: S1 Fig — Correlations within host species between the change in viral load due to coinfection (coinfection viral load—single infection viral load) for CrPV (A) and DCV (B) and the viral loads during coinfection of the opposing virus. Individual points represent the mean viral load or change in viral load at 2 dpi for each DGRP line on a log10 scale, with trend lines added from a univariate least-squares linear model for illustrative purposes. Genetic correlations (r), regression slopes (β), and 95% Cis have been taken from the output of model (1). (DOCX) [file ppat.1011044.s001.docx]

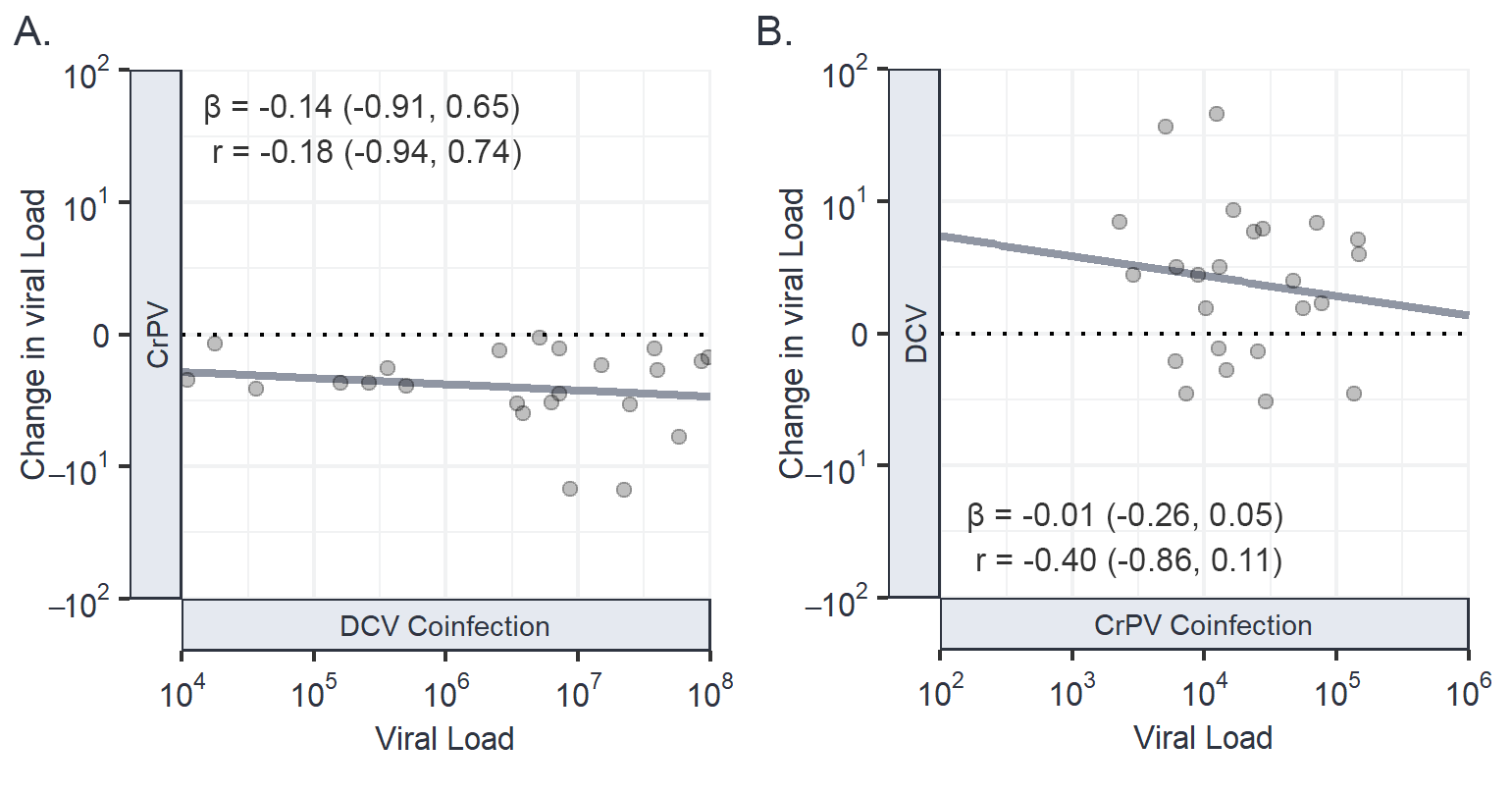


**S1 Fig:** Correlations within host species between the change in viral load due to coinfection (coinfection viral load - single infection viral load) for CrPV (A) and DCV (B) and the viral loads during coinfection of the opposing virus. Individual points represent the mean viral load or change in viral load at 2 dpi for each DGRP line on a log_10_ scale, with trend lines added from a univariate least-squares linear model for illustrative purposes. Genetic correlations (r), regression slopes (β), and 95% Cis have been taken from the output of model (1).
