# Supplementary material for: Investigating the outcomes of virus coinfection within and across host species
Source: PLoS Pathog. 2023 May 22;19(5):e1011044. doi: 10.1371/journal.ppat.1011044 (PMC10237676; doi:10.1371/journal.ppat.1011044)
Supplement: S1 Methods — An additive effect of coinfection was simulated by randomly sampling from normal distributions–generated from the means and variances of changes in viral load across DGRP lines–and adding these values to the single infection viral loads measured across host species. This process was repeated 30 times to produce multiple simulated datasets, which were analysed using phylogenetic GLMMs as described in the main body. The lower 95% credible (HPD) intervals for the estimated changes in viral load during coinfection were examined for each model and consistently showed an ability to detect credible changes in viral load during coinfection of >2-fold across host species. (DOCX) [file ppat.1011044.s003.docx]

# **S1 Methods**

*Power analysis of additive effects of coinfection across host species*

Power analysis was used to determine if the level of replication in the across species experiment was sufficient to detect additive effects of coinfection of similar size and variance to those seen in within-species experiments. An additive effect of coinfection was simulated by randomly sampling from normal distributions – generated from the means and variances of changes in viral load across DGRP lines – and adding these values to the single infection viral loads measured across host species. This process was repeated 30 times to produce multiple simulated datasets, which were analysed using phylogenetic GLMMs as described in the main body. The lower 95% credible (HPD) intervals for the estimated changes in viral load during coinfection were examined for each model and consistently showed an ability to detect credible changes in viral load during coinfection of >2-fold across host species.
