# Supplementary material for: Investigating the outcomes of virus coinfection within and across host species
Source: PLoS Pathog. 2023 May 22;19(5):e1011044. doi: 10.1371/journal.ppat.1011044 (PMC10237676; doi:10.1371/journal.ppat.1011044)
Supplement: S1 Table — Resistant pastrel alleles (G) are coloured orange, and susceptible (A) alleles are coloured blue. (DOCX) [file ppat.1011044.s004.docx]

*S1 Table: DGRP lines*

| **DGRP Line (#)** | **Pastrel Allele** |
| --- | --- |
| 21 | G |
| 59 | G |
| 69 | A |
| 73 | A |
| 109 | G |
| 229 | G |
| 320 | G |
| 358 | G |
| 362 | A |
| 365 | A |
| 386 | G |
| 437 | A |
| 492 | A |
| 502 | G |
| 714 | G |
| 721 | A |
| 738 | G |
| 774 | A |
| 812 | G |
| 820 | A |
| 822 | G |
| 852 | G |
| 855 | G |
| 861 | A |
| 892 | G |

Resistant *pastrel* alleles (G) are coloured orange, and susceptible (A) alleles are coloured blue.
