# Supplementary material for: Investigating the outcomes of virus coinfection within and across host species
Source: PLoS Pathog. 2023 May 22;19(5):e1011044. doi: 10.1371/journal.ppat.1011044 (PMC10237676; doi:10.1371/journal.ppat.1011044)
Supplement: S2 Table — Cycle conditions were as follows: initial denaturation at 95°C for 30 seconds; then 10 cycles of 95°C for 15 seconds, 70–60°C (touchdown Δ of -1°C per cycle) for 30 seconds, and 68°C for 60 seconds; then a final extension at 68°C for 5 minutes. (DOCX) [file ppat.1011044.s005.docx]

*S2 Table: PCR genotyping primers*

| **Primer Name** | **Sequence 5’-3’** |
| --- | --- |
| pst_A2469G(res)_F | GCATGGTGTCCATGAAGAC |
| pst_A2469G(res)_R | TCCTCGACAGGAACCCAGTA |
| pst_G2469A(sus)_F | GCATGGTGTCCATGAAGAT |
| pst_G2469A(sus)_R | CCGATGGCAAAGGATTTTT |

*Cycle conditions were as follows: initial denaturation at 95°C for 30 seconds; then 10 cycles of 95°C for 15 seconds, 70-60°C (touchdown Δ of -1°C per cycle) for 30 seconds, and 68°C for 60 seconds; then a final extension at 68°C for 5 minutes.*
