# Supplementary material for: Investigating the outcomes of virus coinfection within and across host species
Source: PLoS Pathog. 2023 May 22;19(5):e1011044. doi: 10.1371/journal.ppat.1011044 (PMC10237676; doi:10.1371/journal.ppat.1011044)
Supplement: S3 Table — (DOCX) [file ppat.1011044.s006.docx]

*S3 Table: Drosophilidae host species*

| ***Species*** | ***Genus*** | **Wingsize** | **Diet** |
| --- | --- | --- | --- |
| *D. affinis* | *Drosophila* | 1.803 | Malt |
| *D. americana* | *Drosophila* | 2.045 | Malt |
| *D. ananassae* | *Drosophila* | 1.493 | Cornmeal |
| *D. arizonae* | *Drosophila* | 1.548 | Banana |
| *D. baimaii* | *Drosophila* | 1.561 | Cornmeal |
| *D. buzzatii* | *Drosophila* | 1.902 | Malt |
| *D. erecta* | *Drosophila* | 1.581 | Malt* |
| *D. euronotus* | *Drosophila* | 2.222 | Cornmeal |
| *D. flavomontana* | *Drosophila* | 2.192 | Malt* |
| *D. hydei* | *Drosophila* | 2.182 | Cornmeal |
| *D. immigrans* | *Drosophila* | 2.153 | Malt* |
| *D. lacicola* | *Drosophila* | 2.268 | Malt |
| *D. lummei* | *Drosophila* | 2.558 | Malt* |
| *D. mauritiana* | *Drosophila* | 1.507 | Proprionic |
| *D. melanogaster* | *Drosophila* | 1.716 | Cornmeal |
| *D. micromelanica* | *Drosophila* | 1.895 | Cornmeal |
| *D. miranda* | *Drosophila* | 2.395 | Cornmeal |
| *D. mojavensis* | *Drosophila* | 1.650 | Banana |
| *D. montana* | *Drosophila* | 2.706 | Malt* |
| *D. nasuta* | *Drosophila* | 1.917 | Cornmeal |
| *D. nebulosa* | *Drosophila* | 1.826 | Cornmeal |
| *D. obscura* | *Drosophila* | 2.142 | Proprionic |
| *D. paramelanica* | *Drosophila* | 1.946 | Cornmeal |
| *D. persimilis* | *Drosophila* | 2.013 | Malt |
| *D. prosaltans* | *Drosophila* | 1.699 | Proprionic |
| *D. pseudoobscura* | *Drosophila* | 1.863 | Malt |
| *D. putridia* | *Drosophila* | 1.639 | Proprionic |
| *D. saltans* | *Drosophila* | 1.600 | Proprionic |
| *D. santomea* | *Drosophila* | 1.489 | Cornmeal |
| *D. sechellia* | *Drosophila* | 1.424 | Proprionic |
| *D. simulans* | *Drosophila* | 1.484 | Cornmeal |
| *D. sturtevanti* | *Drosophila* | 1.779 | Cornmeal |
| *D. subobscura* | *Drosophila* | 2.056 | Cornmeal |
| *D. sucinea* | *Drosophila* | 1.932 | Cornmeal |
| *D. suzukii* | *Drosophila* | 2.100 | Cornmeal |
| *D. takahashii* | *Drosophila* | 1.559 | Cornmeal |
| *D. teisseri* | *Drosophila* | 1.463 | Cornmeal |
| *D. tropicalis* | *Drosophila* | 1.919 | Cornmeal |
| *D. virilis* | *Drosophila* | 2.253 | Proprionic |
| *D. yakuba* | *Drosophila* | 1.307 | Cornmeal |
| *H. duncani* | *Hirtodrosophila* | 1.969 | Proprionic |
| *S. lativittata* | *Scaptodrosophila* | 1.851 | Banana |
| *S. lebanonensis* | *Scaptodrosophila* | 2.053 | Proprionic |
| *S. pattersoni* | *Scaptodrosophila* | 2.023 | Banana |
| *Z. davidi* | *Zaprionous* | 1.911 | Banana |
| *Z. taronus* | *Zaprionous* | 2.131 | Banana |
| *Z. tuberculatus* | *Zaprionous* | 1.914 | Banana |

* Diets supplemented with additional dry yeast.
