# Supplementary material for: Investigating the outcomes of virus coinfection within and across host species
Source: PLoS Pathog. 2023 May 22;19(5):e1011044. doi: 10.1371/journal.ppat.1011044 (PMC10237676; doi:10.1371/journal.ppat.1011044)
Supplement: S4 Table — Primers were designed to amplify across an intron boundary to ensure only cDNA sequences were amplified. Different primer combinations were used for different host species (see below) to account for SNPs in the primer binding sites. (DOCX) [file ppat.1011044.s007.docx]

*S4 Table: RPL32 primer sequences*

|  | **Name** | **Sequence** |
| --- | --- | --- |
| Forward | RpL32_qPCR_F-a | TGCCAAGTTGTCGCACAAATGG |
|  | RpL32_qPCR_F-b | TGCTAAGTTGTCGCACAAATGG |
|  | RpL32_qPCR_F-c | TGCCAAGCTGTCGCACAAATGG |
|  | RpL32_qPCR_F-d | TGCTAAGCTGTCGCACAAATGG |
|  | RpL32_qPCR_F-e | TGCGAAGTTGTCGCACAAATGG |
|  | RpL32_qPCR_F-f | TGCGAAGCTGTCGCACAAATGG |
| Reverse | RpL32_qPCR_R-a | TGCGCTTGTTGGAACCGTAAC |
|  | RpL32_qPCR_R-b | TGCGCTTGTTGGATCCGTAAC |
|  | RpL32_qPCR_R-c | TGCGCTTGTTGGAACCATAAC |
|  | RpL32_qPCR_R-d | TGCGCTTGTTGGAGCCGTAAC |
|  | RpL32_qPCR_R-e | TGCGCTTGTTAGAACCGTAAC |
|  | RpL32_qPCR_R-f | TACGCTTGTTGGAACCGTAAC |
|  | RpL32_qPCR_R-g | TGCGCTTGTTGGAACCGTAGC |
|  | RpL32_qPCR_R-h | TGCGCTTGTTCGATCCGTAAC |
|  | RpL32_qPCR_R-i | TGCGCTTGTTGGAGCCATAAC |
|  | RpL32_qPCR_R-j | TGCGCTTGTTTGATCCGTAAC |
|  | RpL32_qPCR_R-k | TGCGCTTGTTTGAACCATAAC |
|  | RpL32_qPCR_R-l | TACGCTTGTTGGAACCATAAC |
|  | RpL32_qPCR_R-m | TACGCTTGTTGGAGCCGTAAC |
|  | RpL32_qPCR_R-n | TGCGCTGGTTGGAACCATAAC |
|  | RpL32_qPCR_R-o | TGAGCTTGTTCGATCCGTAAC |
|  | RpL32_qPCR_R-p | TACGCTTGTTGGAGCCATAAC |
|  | RpL32_qPCR_R-q | TGAGCTTGTTTGATCCGTAAC |
|  | RpL32_qPCR_R-r | TAAGCTTGTTGGATCCGTAGC |
|  | RpL32_qPCR_R-s | TCAGCTTGTTGGATCCATAGC |

Primers were designed to amplify across an intron boundary to ensure only cDNA sequences were amplified. Different primer combinations were used for different host species (see below) to account for SNPs in the primer binding sites.
