# Supplementary material for: Investigating the outcomes of virus coinfection within and across host species
Source: PLoS Pathog. 2023 May 22;19(5):e1011044. doi: 10.1371/journal.ppat.1011044 (PMC10237676; doi:10.1371/journal.ppat.1011044)
Supplement: S5 Table — (DOCX) [file ppat.1011044.s008.docx]

*S5 Table: RPL32 primer combinations by Drosophilidae species*

| ***Species*** | **Forward** | **Reverse** |
| --- | --- | --- |
| *D. affinis* | F-a | R-i |
| *D. americana* | F-c | R-a |
| *D. ananassae* | F-f | R-a |
| *D. arizonae* | F-a | R-a |
| *D. baimaii* | F-a | R-r |
| *D. buzzati* | F-a | R-e |
| *D. erecta* | F-d | R-h |
| *D. euronotus* | F-a | R-g |
| *D. flavomontana* | F-c | R-a |
| *D. hydei* | F-a | R-a |
| *D. immigrans* | F-b | R-p |
| *D. lacicola* | F-c | R-a |
| *D. lummei* | F-c | R-a |
| *D. mauritiana* | F-d | R-h |
| *D. melanogaster* | F-d | R-h |
| *D. micromelanica* | F-a | R-g |
| *D. miranda* | F-a | R-d |
| *D. mojavensis* | F-a | R-a |
| *D. montana* | F-c | R-a |
| *D. nasuta* | F-b | R-f |
| *D. nebulosa* | F-b | R-c |
| *D. obscura* | F-b | R-a |
| *D. paramelanica* | F-a | R-g |
| *D. persimilis* | F-a | R-b |
| *D. prosaltans* | F-a | R-n |
| *D. pseudoobscura* | F-a | R-m |
| *D. putridia* | F-d | R-q |
| *D. saltans* | F-a | R-n |
| *D. santomea* | F-a | R-n |
| *D. sechellia* | F-d | R-h |
| *D. simulans* | F-d | R-h |
| *D. sturtevanti* | F-a | R-l |
| *D. subobscura* | F-a | R-i |
| *D. sucinea* | F-b | R-k |
| *D. suzukii* | F-d | R-o |
| *D. takahashii* | F-d | R-o |
| *D. teissieri* | F-d | R-h |
| *D. tropicalis* | F-b | R-k |
| *D. virilis* | F-c | R-a |
| *D. yakuba* | F-d | R-h |
| *H. duncani* | F-f | R-c |
| *S. lativittata* | F-a | R-m |
| *S. lebanonensis* | F-d | R-h |
| *S. pattersoni* | F-a | R-m |
| *Z. davidi* | F-a | R-c |
| *Z. taronus* | F-a | R-c |
| *Z. tuberculatus* | F-a | R-c |
