# Supplementary material for: Investigating the outcomes of virus coinfection within and across host species
Source: PLoS Pathog. 2023 May 22;19(5):e1011044. doi: 10.1371/journal.ppat.1011044 (PMC10237676; doi:10.1371/journal.ppat.1011044)
Supplement: S6 Table — (DOCX) [file ppat.1011044.s009.docx]

*S6 Table: Number of biological replicates (vials of infected flies)*

| **Line/Species** | **CrPV** | **DCV** | **CrPV + DCV** |
| --- | --- | --- | --- |
| DGRP 21 | 12 | 12 | 12 |
| DGRP 59 | 12 | 11 | 12 |
| DGRP 69 | 12 | 11 | 12 |
| DGRP 73 | 12 | 12 | 12 |
| DGRP 109 | 12 | 12 | 10 |
| DGRP 229 | 12 | 12 | 12 |
| DGRP 320 | 12 | 12 | 12 |
| DGRP 358 | 12 | 12 | 11 |
| DGRP 362 | 12 | 11 | 12 |
| DGRP 365 | 12 | 12 | 11 |
| DGRP 386 | 12 | 11 | 11 |
| DGRP 437 | 12 | 12 | 11 |
| DGRP 492 | 12 | 12 | 12 |
| DGRP 502 | 12 | 12 | 12 |
| DGRP 714 | 12 | 12 | 12 |
| DGRP 721 | 11 | 12 | 12 |
| DGRP 738 | 12 | 12 | 12 |
| DGRP 774 | 12 | 12 | 12 |
| DGRP 812 | 12 | 12 | 12 |
| DGRP 820 | 12 | 12 | 12 |
| DGRP 822 | 12 | 12 | 12 |
| DGRP 852 | 12 | 11 | 11 |
| DGRP 855 | 12 | 12 | 12 |
| DGRP 861 | 12 | 12 | 12 |
| DGRP 892 | 12 | 12 | 12 |
| *D. affinis* | 3 | 3 | 3 |
| *D. americana* | 3 | 3 | 2 |
| *D. ananassae* | 3 | 3 | 3 |
| *D. arizonae* | 3 | 3 | 3 |
| *D. baimaii* | 3 | 3 | 3 |
| *D. buzzatii* | 3 | 3 | 3 |
| *D. erecta* | 3 | 3 | 3 |
| *D. euronotus* | 3 | 3 | 3 |
| *D. flavomontana* | 3 | 3 | 3 |
| *D. hydei* | 3 | 3 | 2 |
| *D. immigrans* | 3 | 3 | 3 |
| *D. lacicola* | 3 | 3 | 3 |
| *D. lummei* | 2 | 3 | 2 |
| *D. mauritiana* | 3 | 3 | 3 |
| *D. melanogaster* | 3 | 2 | 3 |
| *D. micromelanica* | 3 | 3 | 3 |
| *D. miranda* | 3 | 3 | 3 |
| *D. mojavensis* | 3 | 2 | 2 |
| *D. montana* | 3 | 3 | 3 |
| *D. nasuta* | 3 | 3 | 3 |
| *D. nebulosa* | 3 | 2 | 3 |
| *D. obscura* | 3 | 3 | 2 |
| *D. paramelanica* | 3 | 3 | 3 |
| *D. persimilis* | 3 | 3 | 3 |
| *D. prosaltans* | 2 | 3 | 3 |
| *D. pseudoobscura* | 3 | 3 | 3 |
| *D. putridia* | 3 | 3 | 3 |
| *D. saltans* | 3 | 3 | 3 |
| *D. santomea* | 3 | 2 | 3 |
| *D. sechellia* | 3 | 3 | 3 |
| *D. simulans* | 3 | 3 | 3 |
| *D. sturtevanti* | 3 | 3 | 2 |
| *D. subobscura* | 3 | 3 | 3 |
| *D. sucinea* | 3 | 3 | 3 |
| *D. suzukii* | 2 | 2 | 2 |
| *D. takahashii* | 3 | 3 | 3 |
| *D. teisseri* | 3 | 3 | 3 |
| *D. tropicalis* | 3 | 3 | 2 |
| *D. virilis* | 2 | 3 | 2 |
| *D. yakuba* | 3 | 3 | 3 |
| *H. duncani* | 3 | 3 | 3 |
| *S. lativittata* | 3 | 3 | 3 |
| *S. lebanonensis* | 3 | 2 | 3 |
| *S. pattersoni* | 3 | 3 | 2 |
| *Z. davidi* | 3 | 3 | 3 |
| *Z. taronus* | 3 | 3 | 3 |
| *Z. tuberculatus* | 3 | 3 | 2 |
