# Supplementary material for: Investigating the outcomes of virus coinfection within and across host species
Source: PLoS Pathog. 2023 May 22;19(5):e1011044. doi: 10.1371/journal.ppat.1011044 (PMC10237676; doi:10.1371/journal.ppat.1011044)
Supplement: S7 Table — Values were taken from model (1), which was fitted on log10-transformed fold-changes in viral load. (DOCX) [file ppat.1011044.s010.docx]

*S7 Table: Heritability (h^2^), coefficients of environmental and additive genetic variation (CV_E_ and CV_A_), and evolvability (I_A_) of viral load for DCV and CrPV during single and coinfection across DGRP lines*

| **Virus** | **Condition** | ***h^2^*** | ***CV_E_*** | ***CV_A_*** | ***I_A_*** |
| --- | --- | --- | --- | --- | --- |
| **DCV** | Single Infection | 0.32 (0.20, 0.46) | 0.24 (0.20, 0.27) | 0.16 (0.12, 0.20) | 0.027 (0.011, 0.046) |
|  | Coinfection | 0.28 (0.16, 0.40) | 0.20 (0.17, 0.23) | 0.13 (0.09, 0.17) | 0.016 (0.008, 0.029) |
| **CrPV** | Single Infection | 0.13 (0.05, 0.22) | 0.21 (0.18, 0.25) | 0.08 (0.05, 0.11) | 0.007 (0.002, 0.012) |
|  | Coinfection | 0.13 (0.06, 0.22) | 0.20 (0.17, 0.24) | 0.08 (0.05, 0.11) | 0.006 (0.002, 0.012) |

Values were taken from model (1), which was fitted on log_10_-transformed fold-changes in viral load.
