# Supplementary material for: Investigating the outcomes of virus coinfection within and across host species
Source: PLoS Pathog. 2023 May 22;19(5):e1011044. doi: 10.1371/journal.ppat.1011044 (PMC10237676; doi:10.1371/journal.ppat.1011044)
Supplement: S8 Table — Values were taken from model (2), which was fitted on log10-transformed Δ fold-changes in viral load. (DOCX) [file ppat.1011044.s011.docx]

*S8 Table: Heritability (h^2^), coefficients of environmental and additive genetic variation (CV_E_ and CV_A_), and evolvability (I_A_) of the change in viral load during coinfection (coinfection - single infection) for DCV and CrPV*

| **Virus** | ***h^2^*** | ***CV_E_*** | ***CV_A_*** | ***I_A_*** |
| --- | --- | --- | --- | --- |
| **DCV** | 0.11 (0.00, 0.23) | 1.34 (0.75, 2.09) | 0.31 (0.00, 0.67) | 0.141 (0.000, 0.444) |
| **CrPV** | 0.02 (0.00, 0.09) | 0.97 (0.58, 1.48) | 0.19 (0.00, 0.48) | 0.060 (0.000, 0.230) |

Values were taken from model (2), which was fitted on log_10_-transformed ∆ fold-changes in viral load.
